# Experimental determination of the light-trapping-induced absorption enhancement factor in DSSC photoanodes

**DOI:** 10.3762/bjnano.6.91

**Published:** 2015-04-02

**Authors:** Serena Gagliardi, Mauro Falconieri

**Affiliations:** 1 ENEA, C. R. Casaccia via Anguillarese 301, 00123 Roma, Italy

**Keywords:** dye-sensitized solar cells, light trapping, optical characterization, photoanode modeling, titania nanostructures

## Abstract

For dye-sensitized solar cells (DSSC), the fundamental process that determines the maximum short-circuit current is the absorption of light. In such devices, this is produced by the concurrent phenomena of light absorption by dye molecules and light trapping in the mesoporous, titania photoanode structure. The decoupling of these two phenomena is important for device characterization and the design of novel photoelectrode geometries with increased optical performance. In this paper, this task is addressed by introducing a spectral absorption enhancement factor as a parameter to quantify the light trapping effect. The experimental value of this parameter was obtained by comparing the experimentally determined fraction of absorbed light by a dye-sensitized photoanode with the light absorbed by the dye without the mesoporous titania structure. In order to gain more insight from this result, the fraction of light absorbed in the photoanode (on the basis of the dye loading capacity of the titania nanospheres) was also calculated by an optical model for the two extreme cases of the absence of light trapping and maximum light trapping. Accordingly, the photocurrent was calculated under the assumption of solar irradiation, which defined two useful boundaries. Using the experimentally derived values of the spectral absorption enhancement factor in the photoanode optical model, the DSSC short-circuit current can be calculated with good agreement with the value measured in practical devices based on the same photoanode structures. Therefore, our approach provides a realistic description of a practical device and can be exploited as an useful tool to assess the optical functionality of novel photoanode structures.

## Introduction

The exploitation of solar irradiation, in particular by the use of photovoltaic (PV) technologies, is a widely recognized target for renewable energy production. Among the different technologies, dye-sensitized solar cells (DSSC) have attracted particular interest, starting from the publication of the seminal paper of Gratzel and O’Reagan in 1991 [1]. A DSSC is a photoelectrochemical system, similar to others studied since the 1960s, based on a dye-sensitized semiconductor. The introduction of a nanostructured, high specific surface area, porous titania electrode was a milestone that finally enabled interesting energy conversion efficiency values, which nowadays are around ≈13% [2]. The use of inexpensive materials and simple manufacturing techniques ensures low-cost production and makes DSSCs a promising class of photovoltaic cells, even though the demonstrated efficiency on the laboratory scale is still well below the performance of more mature photovoltaic technologies. Much effort is devoted to the improvement of the conversion efficiency while maintaining low cost and extending the device lifetime.

One of the key factors for efficient photovoltaic conversion is related to the optical properties of the photoactive material. The knowledge of the optical functionality can therefore aid in the optimization, characterization and design of materials with respect to their applications in PVs as an active layer.

Under the assumption of unitary injection and collection efficiency, the short-circuit photocurrent *J*_SC_ can be written as a function of the optical properties of the device as:

[1]
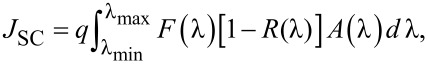


where the integral is over the absorption range of the sensitizing dye, *F*(λ) is the incident photon flux, *R*(λ) is the device reflectivity and *A*(λ) is the fraction of light absorbed in the photoanode (PA).

Therefore, the assessment of the optical functionality of the photoactive layer, that is, the absorbed light fraction, is the starting point to determine one of the key photovoltaic parameters of a device.

In DSSCs, light harvesting occurs at the photoanode, which is comprised of a monolayer of dye adsorbed onto a mesoporous titania film deposited onto a transparent conductive oxide (TCO) layer. The absorption spectrum of the dye determines the light harvesting properties of the cell, if all other optical phenomena are neglected. However, the most commonly used dye, N719, has a low absorption coefficient for red or longer wavelengths [3]. In practice, the nanostructured, porous titania film scatters the light, so that light trapping (LT) phenomenon occurs in the active layer of the cell, resulting in enhanced light absorption and hence the overall improved cell efficiency. The beneficial influence of light scattering in DSSCs was first theoretically analyzed by Usami [4–5] and Ferber [6]. Rothemberger et al. [7] also proposed a careful experimental optical characterization of porous, thick titania electrodes and an optical four flux model to interpret experimental data. All the high efficiency cells reported in the literature exploit LT by both incorporating an additional scattering film [2,8–9] to reflect the light not yet absorbed in the active layer, and by embedding scattering particles in the active layer to increase the light path [10–13]. Nevertheless, light management strategies are usually exploited on the basis of empirical evidence and an accurate systematic approach has not yet been applied.

In this work, the light trapping properties of a PA prepared using standard procedures were experimentally quantified by means of optical spectrophotometry, calculating the light absorbed fraction from reflectivity and transmissivity measurements, and comparing this result with the dye absorption, as obtained by measuring the dye content by desorption analysis. The relevant parameter obtained from such an experimental characterization is the spectral dependence of the absorption enhancement factor resulting from the LT.

Furthermore, the fraction of absorbed light in a PA composed of a mesoscopic structure based on titania nanospheres was also calculated using a simplified, purely optical model in the two extreme cases of no or maximum light trapping [14]. The corresponding short-circuit photocurrent density under AM1.5 illumination of an ideal DSSC was then calculated for the two limiting cases above and compared to that calculated using the experimentally derived absorption enhancement factor. Finally, the measured short-circuit current of a real device fabricated using the optically characterized PA was compared to the predictions of the simplified, purely optical model in the three cases described above.

## Experimental

### Preparation of photoanodes and cell assembly

A standard procedure was used as described in many literature papers [15] using fluorine-doped tin oxide (FTO) glasses, (Pilkinton TEC8^®^, 2 mm thick, cut in 2 × 2 cm^2^ square) as substrates. The photoactive layer was prepared by screen printing Solaronix D/SP titania paste, sensitized with di-tetrabutylammonium cis-bis(isothiocyanato)bis(2,2’-bipyridyl-4,4’-dicarboxylato)ruthenium(II), also known as N719, purchased from Solaronix. Photoanodes of two different sizes were prepared: 0.5 × 0.5 cm^2^ for device assembly, and 1.8 × 1.8 cm^2^ for use in optical characterization. The thickness of the electrodes was 6.5 μm as measured by a mechanical profilometer (Tencor Alphastep).

The PA and the catalyzed cathodes were assembled into a sandwich-type cell and sealed with a 60 μm thick Surlyn^®^ gasket. The electrolyte, a 2 M LiI solution in acetonitrile, 0.22 M I_2_, and 0.5 M 4-*tert*-butylpyridine, was introduced into the cell by syringe vacuum backfilling. All reagents were purchased from Sigma-Aldrich and used without further purification.

### Determination of dye content

The dye load in the PA was measured using a standard literature procedure [16] based on dye desorption in a basic solution and subsequent spectrophotometric quantitative analysis. The dye content was 1.0 × 10^−7^ mol/cm^2^. Calibrated ethanol solutions were also used to obtain values of the spectral molar absorptivity, ε(λ), of the dye.

#### Optical characterization of photoanodes

A double beam UV–vis–NIR spectrophotometer (Perkin Elmer 330) was employed for optical characterization in the spectral range between 400 and 800 nm. A specular reflection attachment, equipped with coated Al mirrors, was used to measure the normal reflectivity, while a BaSO_4_-coated integrating sphere with a diameter of 60 mm was utilized to measure the total transmissivity and the diffuse reflectivity. Collinear and diffuse reflectivity spectra were collected with light incident on the glass side.

#### Cell characterization

*J*–*V* curves were acquired using a Keithley 2420 digital multimeter, under one sun (1000 W/m^2^) AM1.5 illumination, provided by a class A solar simulator (Wacom).

#### Modelling

In the zero-order approximation, the only optical phenomenon occurring in the sensitized titania layer is the light absorption by the dye molecules. In this case, for a given dye composition of known molar absorptivity, the optical properties are univocally determined by the number of molecules adsorbed on the titania electrode. Since only the first dye monolayer adsorbed on the semiconductor surface contributes to the current generation, one can calculate the maximum useful dye load as the number of molecules arranged to form a compact monolayer coating all the available titania surface. The available surface was calculated by representing the PA as a parallelepiped filled with titania spheres. In a simplified scheme, the nanoparticles were assumed to be monodisperse with a fixed packing density (0.64 according to [17]). Then, the total number of adsorbed dye molecules was obtained by dividing the available titania surface by the molecular footprint of the dye (1.6 Å^2^ for the N719 dye [18]). The calculated number of dye molecules on the electrode was then expressed as molarity and used to determine the fraction of light absorbed, according to

[2]
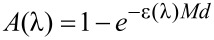


where *M* is the concentration of the dye molecules, *d* the electrode of thickness, and ε(λ) the dye molar absorptivity.

In the first-order approximation, light trapping also occurs in the PA. In order to estimate the fraction of light absorbed in the PA in this case, an enhanced light absorption factor must be considered. Accordingly, we assume that LT can be accounted for by introducing a wavelength-dependent absorption enhancement factor, γ(λ), into Equation 2 as:

[3]
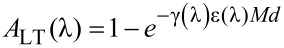


According to Yablonovitch [14], the maximum enhancement factor is expressed by

[4]
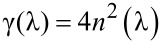


where *n*(λ) is the refractive index of the anatase phase of titania. When the electrode is under real working conditions in the cell, it is immersed in the electrolyte solution that presumably reduces the absorption enhancement due to the LT, partially matching the titania refractive index. Therefore, the maximum enhancement factor calculated in Equation 4 represents an ultimate limit, since it is calculated for the electrode in air.

The short-circuit current density generated by the model PA can be finally calculated using Equation 1, neglecting or taking into account the LT phenomena as described by Equation 2 or Equation 3, respectively.

## Results and Discussion

### Optical characterization

The absorbed light fraction of the fabricated PA was calculated from the experimentally measured values of *R*(λ) and *T*(λ) according to

**Figure 1:**
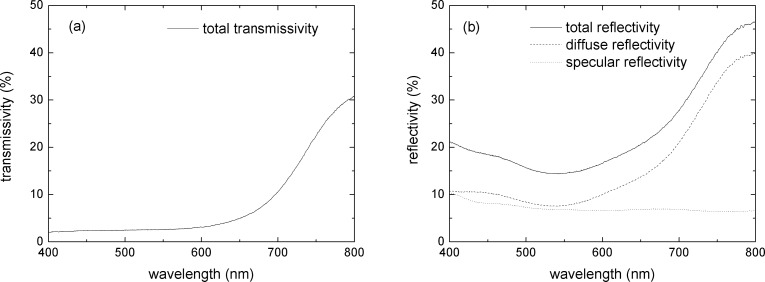
Experimental total transmissivity spectrum (a) and reflectivity spectra (b) of a 6.5 µm thick photoanode.

[5]
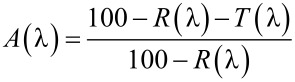


where *R*(λ) and *T*(λ)*,* reported in Figure 1, are the total reflectivity and transmissivity of the PA, respectively. The *R*(λ) is calculated as the sum of the experimentally measured collinear and diffuse reflectivity spectra, also shown in Figure 1.

**Figure 2:**
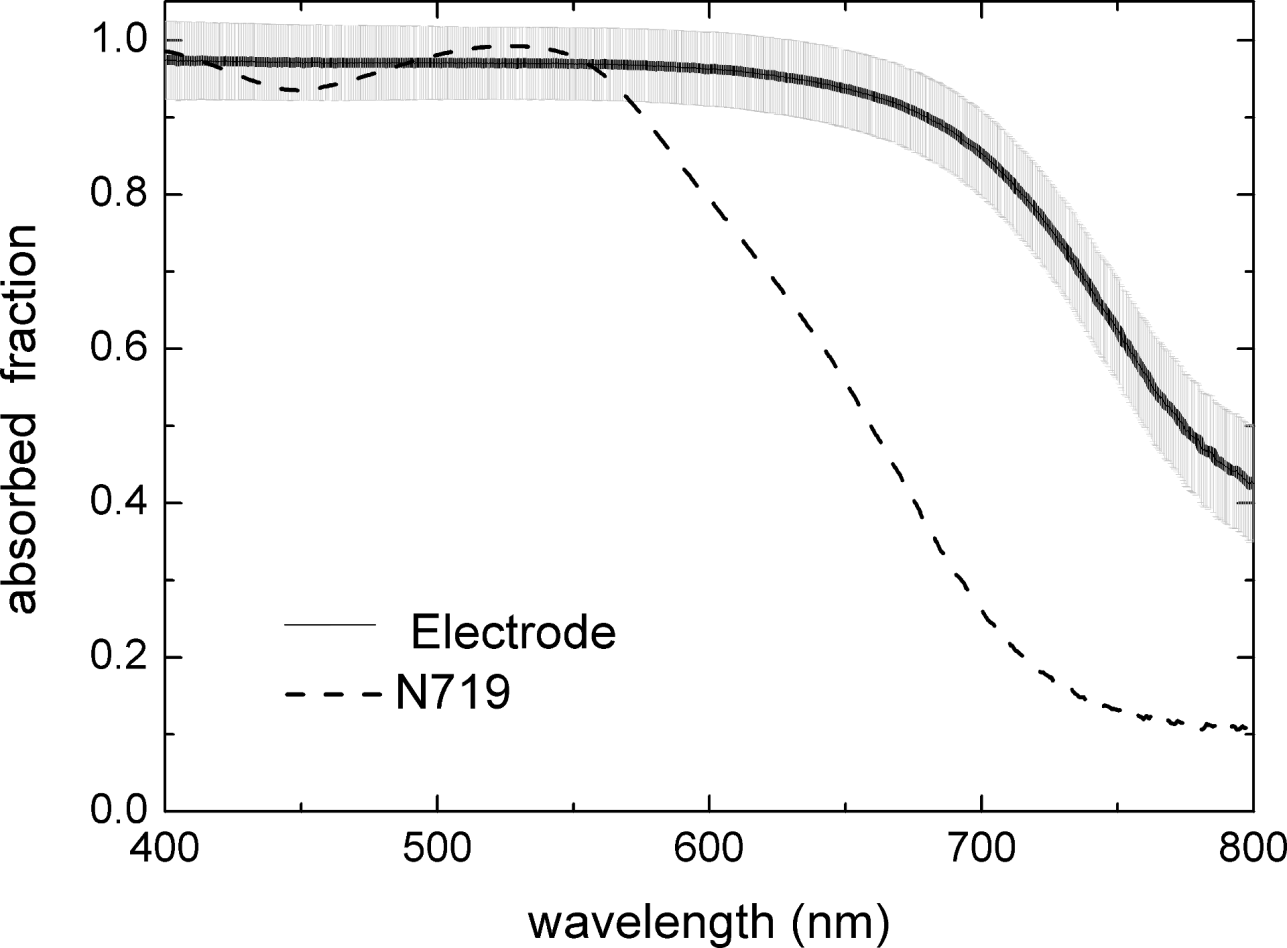
Absorbed light fraction of a PA calculated from experimental reflectivity and transmissivity spectra (continuous line), and of the solution containing the same dye load (dashed line).

In order to measure the amount of light absorbed by the dye that is useful for photovoltaic conversion, the light absorbed in the titania layer and in the FTO was separately measured on a “blank” sample. This sample consisted of a non-diffusive titania layer on FTO, containing the same amount of titania as the sensitized PA. This blank spectrum was subtracted from the data obtained from Equation 5. The resulting value of the absorbed light fraction in the dye of the sensitized PA is reported in Figure 2, where the error statistics from a conventional indetermination of 4% in the measured *T*(λ) values is shown as the grey shaded area. The reason for this uncertainty is that systematic error affects such a low transmissivity in our experiment, which employs a small integrating sphere with highly diffusive samples. In order to reduce the influence of instrumental errors, it can be considered that the absorbed light fraction in the sensitized titania electrode cannot be lower than that of a solution containing the same amount of dye. Therefore, we can safely assume that in the region of strong absorption, the maximum value of the absorbed light fraction in the electrode equals that of the dye. This can be easily calculated according to Equation 2 using the experimentally determined dye content and molar absorptivity.

In order to obtain the light absorption enhancement due to LT, the Naperian logarithm absorbance spectrum of the dye molecules and of the sensitized electrode can be calculated from the respective absorbed light fractions, *A*_N719_(λ) and *A*_Electrode_(λ), as:

[6]
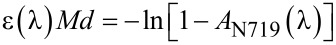


[7]
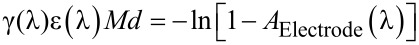


Consequently, the experimental spectrum of the light absorption enhancement γ(λ) in diffusive titania is the given by the ratio between Equation 7 and Equation 6:

[8]
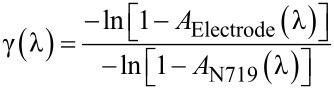


**Figure 3:**
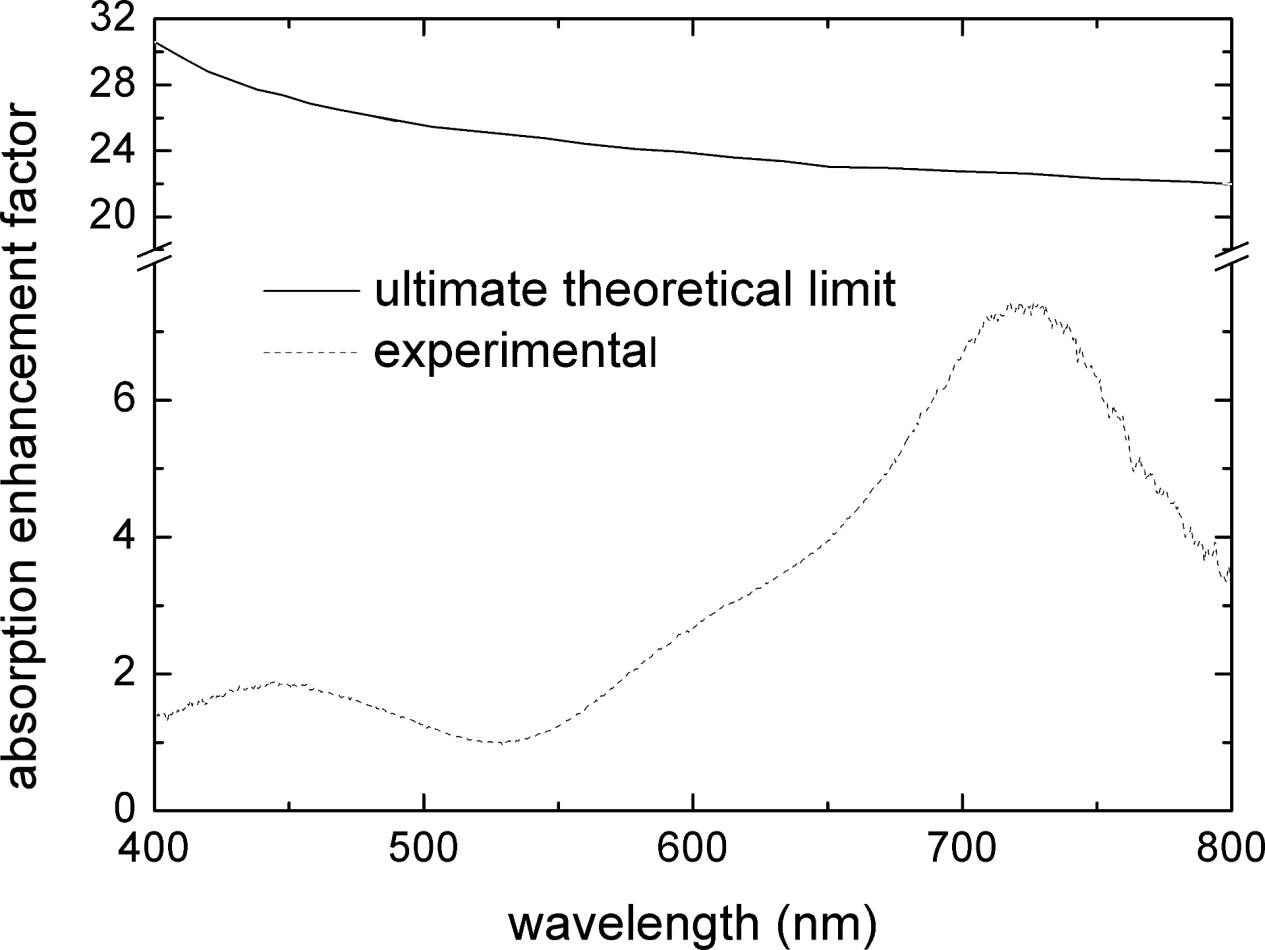
Spectrum of the experimentally determined absorption enhancement factor γ(λ) (dotted line) together with the theoretical maximum value (continuous line).

γ(λ) is reported in Figure 3, together with the ultimate enhancement limit calculated according to [14] with literature values of *n*(λ) [19].

The spectral distribution of the enhancement factor is a characteristic of the mesoporous, nanostructured titania layer, and is mainly related to its morphology and optical properties. In this study, some simplifying assumptions were made by neglecting: (a) the presence of the electrolyte filling the pores, which presumably reduces the LT and (b) the presence of the cathode acting as a back reflector, which therefore enhances the optical absorption. This approach describes a useful, functional characterization for the study of new morphologies of DSSC photoanodes. In fact, it can be applied both to simple structures and layered structures because it considers the photoanode as an effective layer and is based on the comparison of the absorbed light fraction as experimentally measured in the electrode with the absorbance of the dye loaded on the titania.

### Modelling

**Figure 4:**
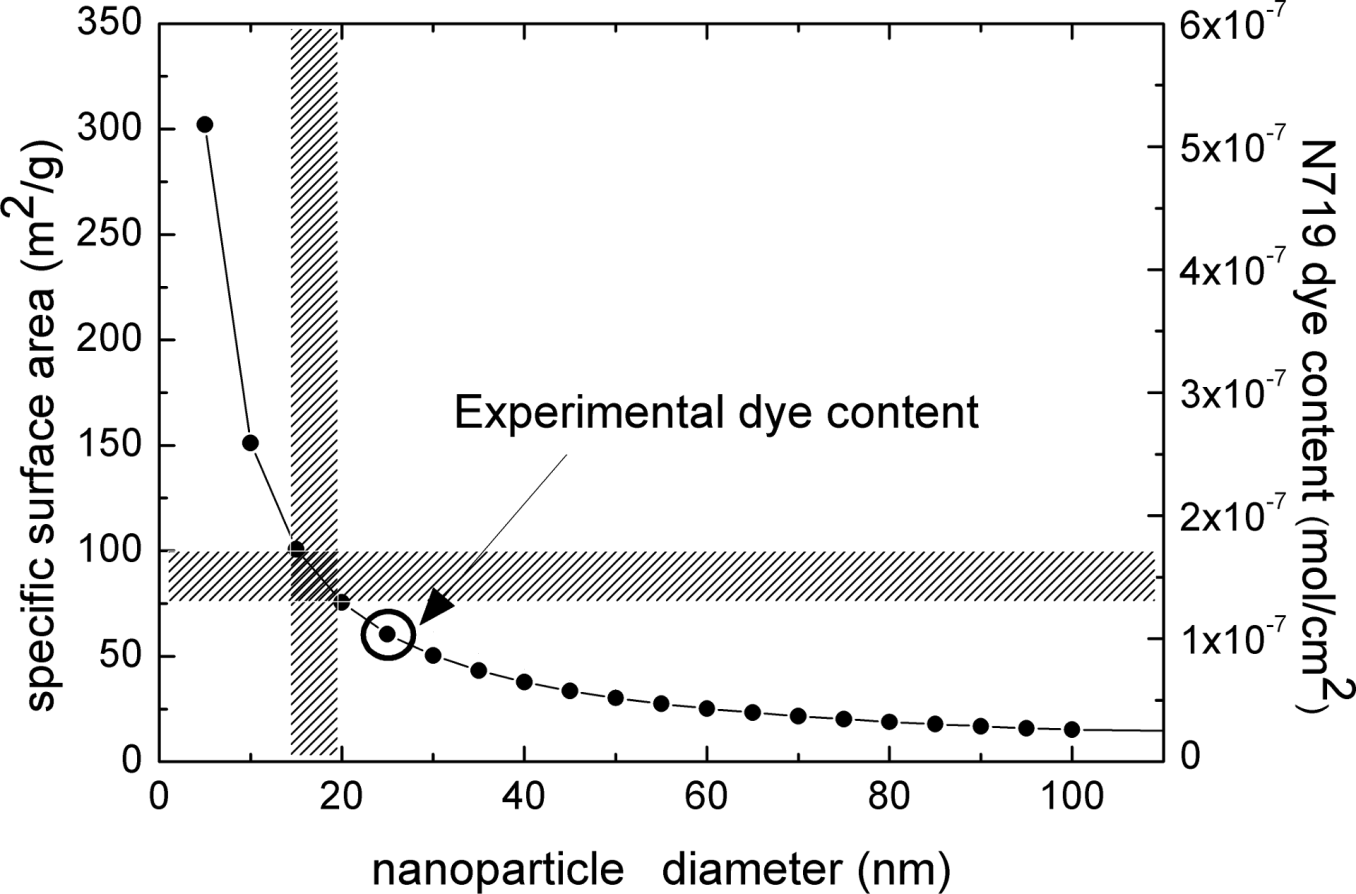
Specific surface area and N719 dye load of the model PA as a function of the nanoparticle diameter. The shadowed region characterizes the uncertainty in the diameter of the nanoparticles in the titania paste used in this work, and the consequent uncertainty on the specific surface area and N719 dye content. The experimental value of the dye load, obtained as reported in the text, is also reported.

The specific surface area and the N719 dye load in a 6.5 μm thick electrode, with a surface area of 1 cm^2^, were obtained using the method described in the Modelling part of the Experimental section and are reported in Figure 4 as a function of the titania nanoparticle diameter.

The nanoparticles contained in the titania paste are 15–20 nm in radius, according to the vendor specifications. This corresponds to an average dye content of (1.5 ± 0.2) × 10^−7^ mol/cm^2^, a value to be compared with the experimentally determined value (equal to 1 × 10^−7^ mol/cm^2^ ) as reported in the Experimental section.

The possible presence of necks between nanoparticles formed in the actual electrode during the sintering process was not taken into account in the model. Thus, the latter provides an upper limit of the anode specific surface. Moreover, the model assumes the entire surface area is coated by a dye monolayer, neglecting the presence of closed pores, which are not accessible to the dye. The presence of necks between nanoparticles and of closed pores can explain the slight disagreement between the experimental and the modeled dye load.

**Figure 5:**
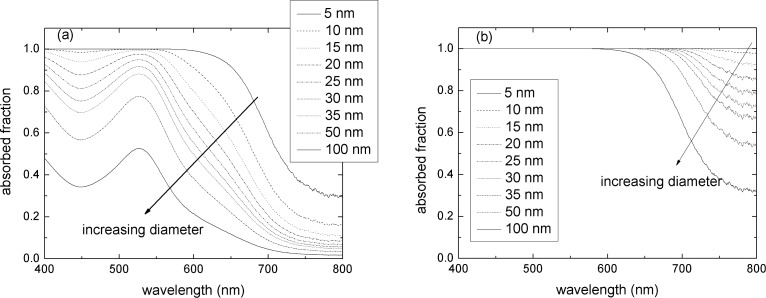
Fraction of light absorbed in the modeled PA in absence of light trapping (a), and in the case of maximum light trapping (b), shown for different diameters of titania nanoparticles.

The fraction of light absorbed in the 6.5 μm × 1 cm^2^ model electrode was calculated using the experimentally determined N719 dye molar absorptivity in Equation 2, or Equation 3 and Equation 4 with literature values of *n*(λ) [19] in absence of LT, or for the ultimate LT respectively. The results are shown in Figure 5a,b, respectively.

Here it is apparent that the larger specific surface area of the smaller nanoparticles allows for loading of a higher number of dye molecules, producing a substantial increase in light absorption. The dramatic effect of light trapping determines the total absorption of light, which occurs well above the region of strong dye absorption, even at low dye loading.

**Figure 6:**
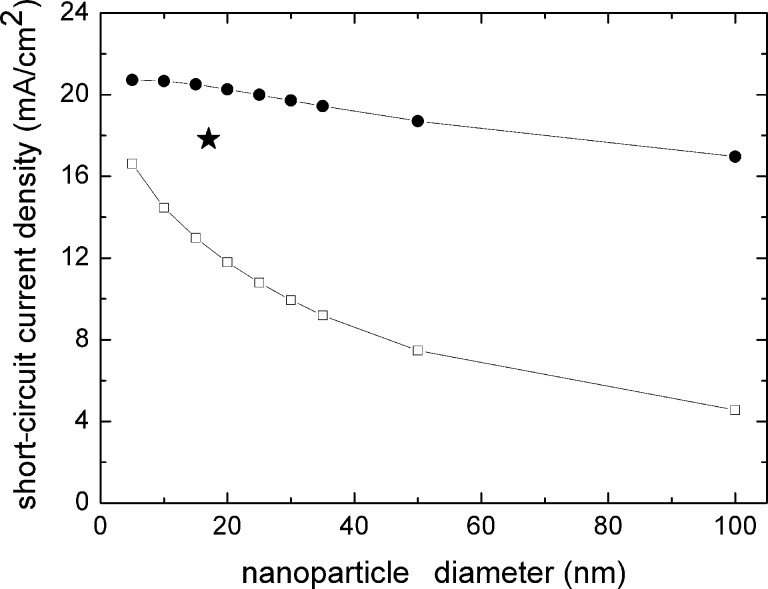
Short-circuit current density calculated for the model electrode, for varying titania nanoparticle size (i.e., varying the dye loading); dark circles: neglecting LT phenomena; open squares: considering the maximum LT. The star represents the value of the current density calculated for the case of experimentally determined light trapping using the average diameter of the TiO_2_ nanoparticles.

The short-circuit current density generated by the model PA was calculated as a function of the titania nanoparticle diameter by using Equation 1 with a AM1.5 photon distribution [20] in the range 400 to 800 nm. The conditions of the absence of LT and the ultimate LT correspond to Equation 2 or Equation 3, and Equation 4, respectively. Here, reflection from the TCO surface was considered to be independent of wavelength over the spectral range of interest and was set equal to 20%, with results shown in Figure 6. The short-circuit current density calculated using Equation 3 with the experimentally determined LT depicted in Figure 3 was found to be 18.3 mA/cm^2^. This is shown in Figure 6 with respect to the nominal average diameter of the TiO_2_ nanoparticles contained in the photoanode fabricated in this work (17.5 nm).

The large differences in the short-circuit current density obtained in the presence or absence of light trapping underlines that (a) considering light diffusion in porous titania electrodes is mandatory both for realistic device modeling and for experimental current to voltage measurement interpretation, and (b) the use of proper light management strategies offers the opportunity of large improvements in the conversion efficiency of the device.

**Figure 7:**
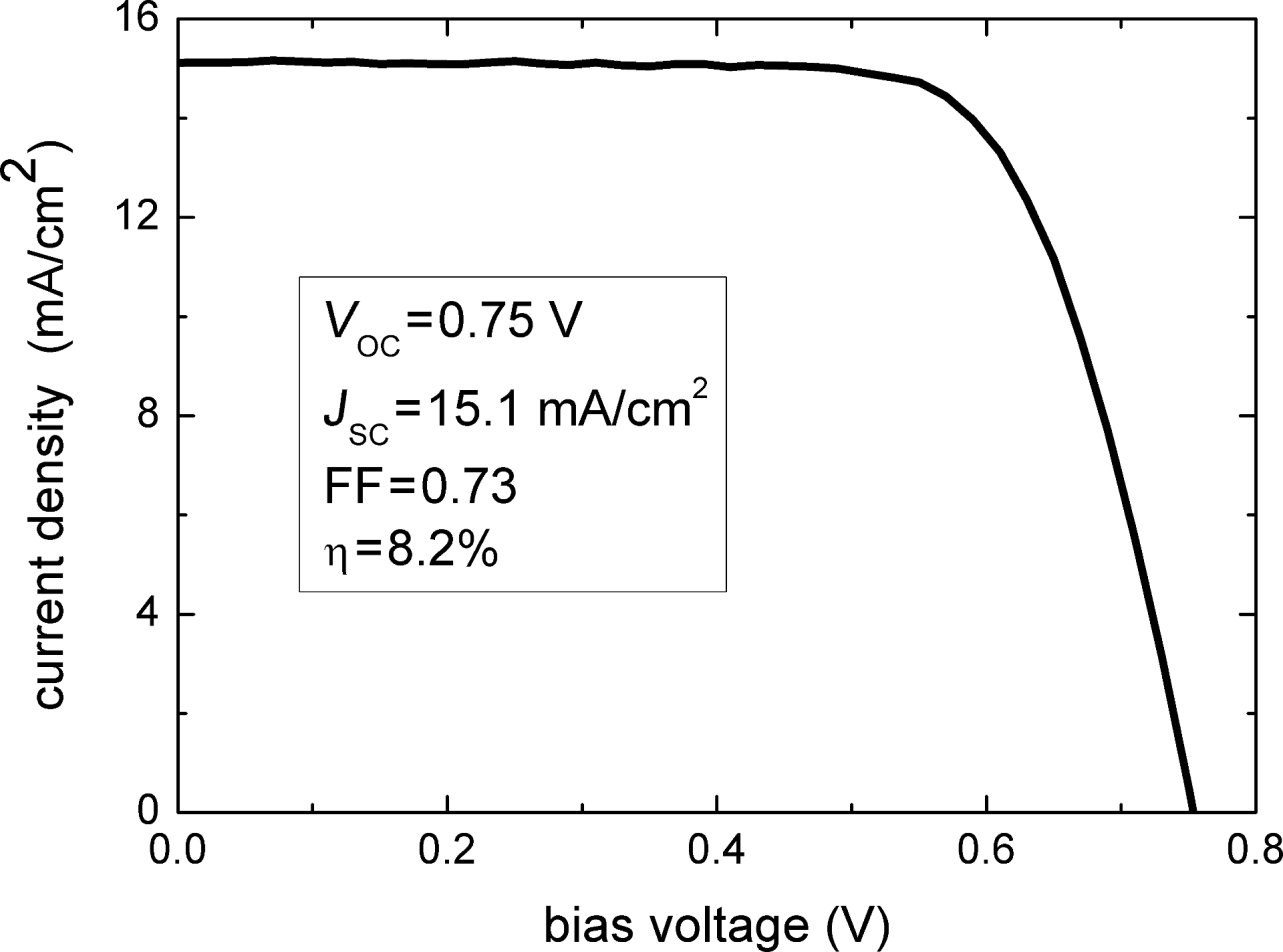
Example of a *J*–*V* characteristic of a DSSC based on the studied PA; the photovoltaic parameters are also reported.

To demonstrate the usefulness of the optical model, it is worth comparing the above results with the corresponding experimental value measured for the real device. Figure 7 shows a typical *J*–*V* characteristic under standard AM1.5 irradiation of a device fabricated using the PA prepared in this work.

The experimental short-circuit current was found to be 15.1 mA/cm^2^, which is in reasonable agreement with the model prediction when taking into account the above mentioned approximations regarding the effect of the electrolyte and the counter-electrode.

## Conclusion

The introduction of a light-trapping-induced absorption enhancement concept has provided a useful tool for modeling and characterizing DSSC photoanodes. The experimental determination of this quantity allowed a rather accurate calculation of the short-circuit current as compared with the experimental values measured in real devices. Comparison with results obtained with a model photoanode both in the absence of and for maximum light trapping values helps to establish the effectiveness of the employed geometry. We can conclude that this tool could be useful for assessment of the optical functionality of novel and possibly layered electrodes in DSSCs.
